# Comparing the association of novel Anthropometric and atherogenicity indices with all-cause, cardiovascular and non-cardiovascular mortality in a general population of Iranian adults

**DOI:** 10.1016/j.ajpc.2025.100936

**Published:** 2025-01-27

**Authors:** Parisa Hajihashemi, Noushin Mohammadifard, Motahare Bateni, Fahimeh Haghighatdoost, Maryam Boshtam, Jamshid Najafian, Masoumeh Sadeghi, Niloufar Shabani, Nizal Sarrafzadegan

**Affiliations:** a Isfahan Gastroenterology and Hepatology Research Center, Isfahan University of Medical Sciences, Isfahan, Iran; b Interventional Cardiology Research Center, Cardiovascular Research Institute, Isfahan University of Medical Sciences, Isfahan, Iran; c Isfahan Cardiovascular Research Center, Cardiovascular Research Institute, Isfahan University of Medical Sciences, Isfahan, Iran; d Hypertension Research Center, Cardiovascular Research Institute, Isfahan University of Medical Sciences, Isfahan, Iran; e Cardiac Rehabilitation Research Center, Cardiovascular Research Institute, Isfahan University of Medical Sciences, Isfahan, Iran

**Keywords:** Cardiovascular, Mortality, Atherogenic index, Anthropometric index

## Abstract

**Background:**

The association of novel anthropometrics and novel atherogenicity indices with mortality remains uncertain.

**Objective:**

To compare the association of novel anthropometrics and atherogenicity indices with all-cause, cardiovascular (CVD), and non-CVD mortality in Iranian adults.

**Methods:**

Utilizing data from Isfahan Cohort Study, 5432 participants aged older than 35 years were enrolled. Three anthropometrics indices including a body shape index (ABSI), body roundness index (BRI) and abdominal volume index (AVI), and three atherogenicity indices including atherogenic index of plasma (AIP), Castelli risk index (CRI) and the cholesterol index (CI) were calculated. Cox proportional hazards regression models were used to explore the association between indices and mortality.

**Results:**

After a median follow-up of 11.25 years, the ABSI was independently associated with increased risk of all-cause mortality (HR_Q4__vs. Q1_ = 1.43, 95 % CI: 1.07, 1.92; P trend = 0.02). A positive, independent association was also observed between CRI-II (HR_Q4__vs. Q1_ = 1.49, 95 % CI: 0.99, 2.25; P trend = 0.04) and AIP (HR_Q4__vs. Q1_ = 1.81, 95 % CI: 1.92, 2.27; P trend = 0.01) and CVD mortality. For non-CVD mortality, despite a direct link for ABSI (HR_Q4__vs. Q1_ = 1.92, 95 % CI: 1.32, 2.80; P trend = 0.001), an inverse association was found for CI (HR_Q4__vs. Q1_ = 0.68, 95 % CI: 0.49, 0.95; P trend = 0.007).

**Conclusion:**

Amongst various investigated anthropometric indices, ABSI was directly related to all-cause and non-CVD mortality. However, atherogenicity indices including CRI-II and AIP could predict the incidence risk of CVD mortality among Iranians. Further studies are warranted to confirm these findings.

## Graphical abstract

**Figure:**
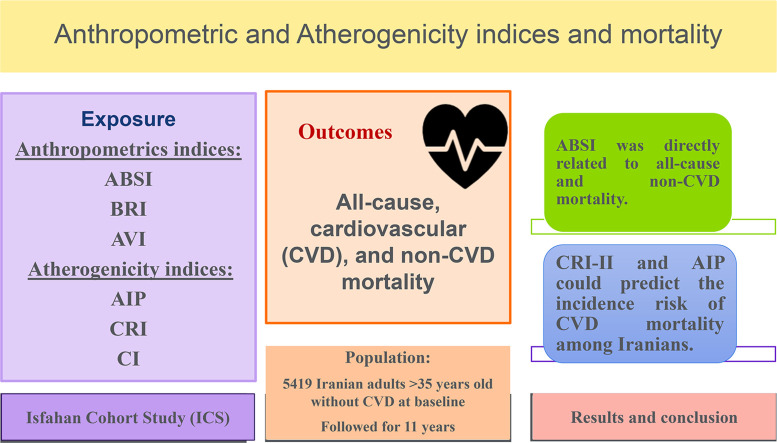

## 1 Introduction

During the era of epidemiologic transition, the communicable infectious diseases have been replaced by non-communicable chronic diseases (NCDs) [1]. NCDs, accounting for nearly two-thirds of the world's deaths, represent an urgent public health challenge [1]. Cardiovascular disease (CVD) remains the predominant cause of mortality in both high-income and low- and middle-income countries [2]. Modifiable risk factors such as cardiometabolic, behavioral and environmental risk factors are among major drivers of CVD pathogenesis and progression [2]. As a result, CVD and its health consequences can be delayed by prevention and alleviation of modifiable risk factors [3]. Among these risk factors, obesity and dyslipidemia have been sparingly studied [[4], [5], [6]].

Epidemiological studies have mostly considered high body mass index (BMI) as the index of obesity [7]. However, BMI has several flaws that make it an imperfect measure of body fat, including its inability to distinguish between muscles mass and fat mass and reflecting fat locations [7]. Recent studies suggested that besides overall obesity, the distribution of body fat is an important risk factor for cardiometabolic outcomes [8,9]. To compensate for these limitations, novel anthropometric indices such as A Body Shape Index (ABSI), Body Roundness Index (BRI), and abdominal volume index (AVI), which measures abdominal obesity and predominantly visceral fat, have been developed [[10], [11], [12]]. Previous studies have revealed that higher ABSI and BRI were positively linked to a greater fraction of visceral fat and thus a higher risk of CVD and mortality [11,[13], [14], [15]].

Dyslipidemia is defined by abnormal levels of total cholesterol (TC), triglycerides (TG), low-density lipoprotein cholesterol (LDL-C), and high-density lipoprotein cholesterol (HDL-C), which promotes atherosclerosis [6]. Comprehensive lipid ratios have been shown to be better predictors of coronary artery disease (CAD) rather than individual lipid values. In this context, novel indices, such as the atherogenic index of plasma (AIP), Castelli risk indexes 1 (CRI-1) and 2 (CRI-2) and the cholesterol index (CI) have been indicated as independent predictor factor for CAD [16].

Identifying subjects at high risk of CVD and accompanying mortality in its early stages needs much attention. Since dyslipidemia and obesity are the major modifiable risk factors of CVD outcomes [6,17,18], exploring the potential of the anthropometrics and dyslipidemia indicators to predict risk of CVD mortality will improve our knowledge regarding early diagnosis and management of CVD and coexist mortality. However, despite the importance of this urgent issue, there is a paucity of evidence on the link between novel anthropometrics and atherogenicity indices and the risk of CVD, non-CVD and all-cause mortality. Therefore, the purpose of this study was to determine whether higher anthropometrics (ABSI, BRI, AVI), and atherogenicity indices (AIP, CRI and CI) can predict the incidence risk of CVD, non-CVD and all-cause mortality in the general Iranian population.

## 2 Methods and materials

**Study population:** We conducted the analysis in Isfahan cohort study (ICS), a dynamic prospective cohort study established in 2001 in three districts located in the central of Iran (Isfahan, Najaf-Abad, and Arak) [19]. The methodology of ICS has already been detailed [19]. This study protocol was reviewed and approved by the Ethics Committee of the Research Council of Isfahan Cardiovascular Research Center, a World Health Organization collaborating center in Isfahan, Iran (#82,112). For the current analysis, individuals with a history of CVDs (*n* = 181), and missing data on CVD follow-up or those lost to follow-up (*n* = 891) were excluded. After exclusions, a total number of 5432 subjects remained in this study.

**Data collection:** Information on demographic and socioeconomic variables (age, sex, educational level, and marital status), lifestyle aspects such as smoking status and physical activity level and medical history was collected with an interviewer-administered questionnaire at baseline [[20], [21], [22]]. The validity of physical activity questionnaire has been evaluated previously [20].

**Biochemical assessments:** 12-hour overnight fasting blood samples were collected to measure the levels of fasting blood sugar (FBS) and serum lipids including triglyceride (TG), total cholesterol (TC), high-density lipoprotein cholesterol (HDL-C), and low-density lipoprotein cholesterol (LDL-C). Serum lipids levels were determined using commercially available enzymatic reagents (Pars Azmoun, Iran) with an automatic device (Selecta E, Vitalab, Netherlands).

### 2.1 Novel anthropometrics indices (ABSI, BRI and AVI) assessment

Height was measured to the nearest 0.5 cm using a nonelastic meter while participants were barefoot and standing in a normal position. Weight was measured on a scale to the nearest 100 g while participants were in light clothing. Waist circumference (WC) was measured at a level midway between the lower rib margin and the iliac crest using a tape horizontally fixed around the body. BMI was calculated by dividing weight (kg) to height (m2). Hip circumference was measured at the point of maximum circumference over the buttocks using a nonelastic meter.

ABSI, BRI, and AVI were computed by previously mentioned formulas [23] using WC (m), BMI (kg/m2), and height (m) as below:

**ABSI** = $\frac{\text{wc}}{BMI{\;}^{\frac{2}{3}}\;\times \;height{\;}^{\frac{1}{2}}}$

**BRI** = 364.2 + 365.5 × $\sqrt{1-(\frac{(\frac{WC}{2\pi })2}{(0.5\;height)2})}$

**xAVI** = $\frac{2\;{\left(\text{WC}\right)}^{2}\;+\;0.7\;{\left(\text{WC}-\text{hip}\right)}^{2}}{1000}$

### 2.2 Novel atherogenicity indices assessment

Atherogenicity indices (AIP, CRI, and CI) were calculated by using LDL-C, HDL-C, and TG using following formula [23]:

**CRI-1** = $\frac{TC}{HDL-C}$

**CRI-2** = $\frac{LDL-C}{HDL-C}$

**AIP** = log $\frac{\text{TG}}{HDL-C}$

**CI** = IF TG > 400 (LDL – HDL + 1.5) × TG

**CI** = IF TG < 400 (LDL – HDL) × TG

**Ascertainment of deaths:** Totally 487 all-cause death, 191 CVD death, and 296 non-CVD death occurred after 11.25 y of follow-up. To obtain information on participants’ deaths, we took the approach of verbal autopsy from surviving close family members using a structured primary interview in which the first question was ‘is he/she alive?”.

### 2.3 Statistical analysis

Based on the occurrence of outcome (died or survived), general characteristics of participants at recruitment were compared either using Chi-square test for categorical variables or independent sample *t*-test for continuous variables. Continuous and categorical variables were reported as mean ± SD and number and percent, respectively. Using the date of recruitment and the date of death or last follow-up, which ever occurred first, person-years of follow-up was calculated. Crude and multivariate-adjusted hazard ratios (HRs) and 95 % CI for association between anthropometric and atherogenicity indices and mortality from all causes, CVD and other than CVD were estimated using Cox proportional hazards regression. Confounding factors were controlled in several multivariate models. Model 1 was controlled for age at baseline and sex. Model 2 was additionally adjusted for marital status, education level, and lifestyle factors including smoking status and total daily physical activity. All analyses were performed using SPSS (v. 26) and STATA (v 14.0), and P values < 0.05 were considered statistically significant.

## 3 Results

The study consisted of 5432 individuals, of which 2647 were males (48.7 %). The mean±SD age of the participants was 50.7 ± 11.6. We identified a total number of 447 all-cause mortality (overall incidence of 6.88 per 1000 person-years), including 172 CVD mortality (overall incidence of 2.68 per 1000 person-years) and 275 non-CVD mortality (overall incidence of 4.19 per 1000 person-years) during a follow-up duration of 11.25 years.

The general characteristics of participants are illustrated in Table 1**.** Individuals with all-cause, CVD, and non-CVD mortality were older (P value<0.001), less educated (P value<0.001), had lower level of physical activity (P value<0.001), and were more likely to be smoker (P value<0.05) compared to individuals who survived. In addition, HTN (P value<0.001) and T2DM (P value<0.05) were more frequent in individuals with all-cause, CVD, and non-CVD mortality compared to those who survived. However, overweight was more frequent in individuals who died from all and non-CVD causes (P value<0.001), while higher levels of TG (P value = 0.02), LDL (P value = 0.01), and total cholesterol (P value = 0.009) were more frequent only in individuals who died due to CVD. The medians (IQR) of the quartiles of anthropometric and atherogenicity indices are reported in Table 2**.**

**Table 1:** Baseline characterizes of participants by all-cause mortality, CVD-mortality, and non-CVD mortality.

	All-cause mortality			CVD mortality			Non-CVD mortality		
	Death	Alive	P _value_*	Death	Alive	P _value_*	Death	Alive	P _value_*
Men, n (%)	277 (57.3)	2458 (48.2)	<0.001	114(4.2)	2621(95.8)	<0.001	163(55.3)	2572(48.6)	0.02
Age, years (Mean±SD)	63.9 ± 11.4	49.7 ± 10.9	<0.001	64.33 ± 11.14	50.49 ± 11.45	<0.001	63.64 ± 11.6	50.24 ± 11.3	<0.001
Education, n (%)			<0.001			<0.001			<0.001
≤5 years	428(89)	3551(69.7)		166(88.8)	3813(70.7)		262(89.1)	3717(70.4)	
6–12 years	47(9.8)	1213(23.8)		19(10.2)	1241(23)		28(9.5)	1232(23.3)	
≥13 years	6(1.2)	332(6.5)		2(1.1)	336(6.2)		4(1.4)	334(6.3)	
Married, n (%)	398(82.4)	4679(91.7)	<0.001	163(86.7)	4941(91.1)	0.04	235(79.7)	4842(91.5)	<0.001
Physical activity (MET.min/ week)	662.31 ± 579.2	883.89 ± 542.3	<0.001	600.61 ± 508.3	873.9 ± 548.2	<0.001	701.62 ± 617.8	873.83 ± 543.6	<0.001
Current smokers, n (%)	76(15.8)	832(16.3)	0.001	34(18.1)	874(16.2)	0.03	42(14.3)	866(16.4)	0.01
Hypertension, (n%)	254(52.6)	1414(27.7)	<0.001	119(63.3)	1549(28.7)	<0.001	135(45.8)	1533(29)	<0.001
Diabetes, n (%)	105(21.7)	530(10.4)	<0.001	60(31.9)	575(10.7)	<0.001	45(15.3)	590(11.2)	0.03
Dyslipidemia, n (%)	410(84.9)	4465(87.5)	0.09	167(88.8)	4708(87.2)	0.51	243(82.4)	4632(87.6)	0.009
BMI (Kg/m^2^)	25.81 ± 4.39	26.78 ± 4.45	<0.001	26.34 ± 4.44	26.71 ± 4.45	0.26	25.47 ± 4.34	26.77 ± 4.45	<0.001
Waist circumference (cm)	95.33 ± 12.7	94.72 ± 12.2	0.29	96.05 ± 12.96	94.73 ± 12.28	0.15	94.88 ± 12.65	94.77 ± 12.29	0.88
TC, mg/dl	216.08 ± 53.8	214.32 ± 52.32	0.48	224.32 ± 57.01	214.13 ± 52.2	0.009	210.83 ± 51.16	214.68 ± 52.35	0.22
LDL, mg/dl	130.56 ± 43.85	128.94 ± 43.51	0.43	136.81 ± 46.35	128.81 ± 43.2	0.01	126.58 ± 41.78	129.22 ± 43.64	0.31
HDL, mg/dl	46.8 ± 10.1	46.9 ± 10.3	0.83	45.73 ± 9.74	46.98 ± 10.39	0.10	47.55 ± 10.43	46.90 ± 10.37	0.29
TG, mg/dl	193.34 ± 106.51	192.17 ± 103.84	0.81	208.83 ± 117.1	191.69 ± 103.54	0.02	183.48 ± 98.11	192.76 ± 104.37	0.13
FBS, mg/dl	100.00 ± 49.34	87.66 ± 30.74	<0.001	111.50 ± 60.26	87.93 ± 31.28	<0.001	92.81 ± 39.53	88.45 ± 32.44	0.03
ABSI	0.0036 ± 0.001	0.0034 ± 0.001	<0.001	0.0035 ± 0.001	0.0034 ± .001	0.53	0.0037 ± 0.001	0.0034 ± 0.001	<0.001
BRI	5.508 ± 2.00	5.270 ± 1.92	0.012	5.697 ± 2.133	5.276 ± 1.922	0.005	5.391 ± 1.91	5.284 ± 1.93	0.37
AVI	18.601 ± 4.77	18.293 ± 4.58	0.17	18.962 ± 5.050	18.298 ± 4.583	0.062	18.37 ± 4.59	18.31 ± 4.60	0.83
AIP	0.568 ± .238	0.567 ± 0.242	0.88	0.614 ± 0.240	0.565 ± 0.241	0.009	0.540 ± 0.23	0.568 ± 0.24	0.06
CRI, II	2.890 ± 1.11	2.870 ± 1.13	0.70	3.092 ± 1.193	2.862 ± 1.132	0.009	2.762 ± 1.05	2.875 ± 1.14	0.10
CI	17,105.81 ± 1567.14	16,405.29 ± 14,763.79	0.34	20,411.87 ± 19,983.95	16,333.75 ± 14,625.88	<0.001	15,038.02 ± 11,800.78	16,539.02 ± 14,982.17	0.10

Data reported are mean ± SD.

*Chi-square test was used for categorical variables and independent *t*-test for continuous variables.

BMI: Body Mass Index, LDL: low-density lipoprotein, HDL: high-density lipoprotein, TC: total cholesterol, TG: triglyceride.

**Table 2:** The median (IQR) of the quartiles of anthropometric and atherogenicity indices.

	Quartiles of indices			
Variables	Q1	Q2	Q3	Q4
**Anthropometric indices**				
ABSI	0.0024 (0.0021–0.0026)	0.0030 (0.0029–0.0032)	0.0036 (0.0034–0.0038)	0.0045 (0.0042–0.0051)
BRI	3.09 (2.56–3.52)	4.53 (4.23–4.80)	5.70 (5.37–6.01)	7.51 (6.88–8.50)
AVI	12.90 (11.41–13.92)	16.59 (15.89–17.33)	19.60 (18.82–20.10)	23.34 (22.07–25.54)
**Atherogenicity indices**				
AIP	0.28 (0.19–0.34)	0.48 (0.44–0.52)	0.64 (0.60–0.68)	0.85 (0.78–0.95)
CRI&II	1.65 (1.36–1.88)	2.41 (2.24–2.58)	3.08 (2.90–3.28)	4.13 (3.80–4.75)
CI	4268 (2336–5722)	9731 (8308.64–11,100)	16,446.5 (14,396–18,619.5)	30,660 (25,284.5–40,679.6)

Data reported are median (IQR).

ABSI: a body shape index, BRI: body roundness index, AVI: abdominal volume index, AIP: atherogenic index of plasma, CRI: Castelli risk index, and CI: cholesterol index.

Table 3 displays the multivariable-adjusted HRs for all-cause, CVD, and non-CVD mortality across quartiles of novel anthropometric indices. For all-cause mortality, only ABSI was associated with higher incidence risk of mortality either in crude (HR_Q4__vs. Q1_ = 2.00, 95 % CI: 1.52–2.62, P trend<0.001) or in fully-adjusted model (HR_Q4__vs. Q1_ = 1.43, 95 % CI: 1.07–1.92, P trend = 0.02). For CVD mortality, only ABSI in the crude, but not adjusted model, was associated with elevated risk (HR_Q4__vs. Q1_ = 1.46, 95 % CI: 0.82–1.95, P trend = 0.04). Regarding non-CVD mortality, higher ABSI scores were associated with increased incidence risk of mortality in both crude (HR_Q4__vs. Q1_ = 2.44, 95 % CI:1.71–3.48, P trend<0.001) and adjusted (HR_Q4__vs. Q1_ = 1.92, 95 % CI: 1.32–2.80, P trend = 0.001) models. AVI and BRI were not associated with increased risk of all-cause, CVD, and non-CVD mortality in any of the crude and adjusted models.

**Table 3:** Multivariable adjusted HRs for all-cause, CVD, and non-CVD mortality across quartiles of novel anthropometric indices.

	Case/person years	Model 1	Model 2	Model 3
All-cause mortality				
**ABSI**				
Q1	185/177,632	1	1	1
Q2	132/183,038	1.41 (1.06–1.88)	1.31 (0.98–1.74)	1.35 (1.01–1.80)
Q3	106/171,143	1.73 (1.31–2.29)	1.43 (1.07–1.91)	1.43 (1.07–1.91)
Q4	92/168,426	2.00 (1.52–2.62)	1.45 (1.08–1.93)	1.43 (1.07–1.92)
**P trend^1^**		<0.001	0.01	0.02
**AVI**				
Q1	114/159,548.9	1	1	1
Q2	127/175,899.9	0.89 (0.68–1.15)	0.85 (0.65–1.10)	0.88 (0.68–1.15)
Q3	130/177,967.1	0.88 (0.68–1.13)	0.79 (0.61–1.02)	0.84 (0.65–1.09)
Q4	114/186,824	0.99 (0.77–1.27)	0.93 (0.72–1.20)	0.96 (0.75–1.24)
**P trend^1^**		0.97	0.56	0.77
**BRI**				
Q1	131/164,565	1	1	1
Q2	122/173,861	1.07 (0.82–1.39)	0.99 (0.76–1.29)	0.99 (0.76–1.30)
Q3	123/180,353	1.05 (0.81–1.37)	0.90 (0.68–1.17)	0.93 (0.71–1.22)
Q4	111/181,460	1.18 (0.91–1.52)	0.94 (0.71–1.25)	0.95 (0.71–1.26)
**P trend^1^**		0.23	0.56	0.65
**CVD mortality**				
**ABSI**				
Q1	411/177,632	1	1	1
Q2	324/183,038	1.26 (0.82–1.95)	1.11 (0.71–1.72)	1.14 (0.73–1.77)
Q3	259/171,143	1.58 (1.04–2.41)	1.19 (0.77–1.85)	1.17 (0.75–1.82)
Q4	281/168,426	1.46 (0.95–2.24)	0.93 (0.59–1.47)	0.89 (0.56–1.41)
**P trend^1^**		0.05	0.75	0.56
**AVI**				
Q1	316/159,548	1	1	1
Q2	326/175,899	0.97 (0.63–1.47)	0.93 (0.61–1.42)	0.96 (0.63–1.47)
Q3	297/177,967	1.06 (0.70–1.60)	0.97 (0.64–1.47)	1.04 (0.68–1.57)
Q4	305/186,824	1.03 (0.68–1.55)	1.00 (0.66–1.52)	1.03 (0.67–1.57)
**P trend^1^**		0.77	0.90	0.79
**BRI**				
Q1	361/164,565	1	1	1
Q2	337/173,861	1.07 (0.69–1.65)	1.00 (0.64–1.54)	0.98 (0.63–1.52)
Q3	278/180,353	1.29 (0.85–1.95)	1.15 (0.75–1.77)	1.19 (0.77–1.82)
Q4	285/181,460	1.25 (0.83–1.91)	1.12 (0.71–1.77)	1.12 (0.71–1.77)
**P trend^1^**		0.19	0.50	0.47
**Non-CVD mortality**				
**ABSI**				
Q1	336/177,632	1	1	1
Q2	221/183,038	1.54 (1.05–2.24)	1.46 (1.00–2.14)	1.51 (1.03–2.21)
Q3	181/171,143	1.86 (1.28–2.69)	1.62 (1.11–2.38)	1.63 (1.11–2.39)
Q4	137/168,426	2.44 (1.71–3.48)	1.91 (1.32–2.79)	1.92 (1.32–2.80)
**P trend^1^**		<0.001	0.001	0.001
**AVI**				
Q1	177/159,548			
Q2	209/175,899	0.84 (0.61–1.17)	0.80 (0.57–1.11)	0.83 (0.60–1.16)
Q3	232/177,967	0.77 (0.55–1.08)	0.69 (0.49–0.97)	0.73 (0.52–1.03)
Q4	183/186,824	0.96 (0.71–1.32)	0.89 (0.65–1.22)	0.93 (0.67–1.28)
**P trend^1^**		0.79	0.41	0.57
**BRI**				
Q1	205/164,565	1	1	1
Q2	191/173,861	1.07 (0.77–1.49)	0.98 (0.71–1.37)	1.00 (0.72–1.40)
Q3	221/180,353	0.92 (0.66–1.29)	0.76 (0.53–1.07)	0.79 (0.55–1.12)
Q4	182/181,460	1.13 (0.82–1.56)	0.84 (0.59–1.20)	0.86 (0.60–1.23)
**P trend^1^**		0.62	0.21	0.25

1 Derived from Mantel-Haenszel.

Model 1: Crude.

Model 2: Adjusted for age and sex.

Model 3: Additionally adjusted for smoking status, total daily physical activity, marital status, education level.

ABSI: a body shape index, AVI: abdominal volume index, and BRI: body roundness index.

Table 4 illustrates the multivariable-adjusted HRs for all-cause, CVD, and non-CVD mortality across quartiles of atherogenicity indices. For all-cause mortality, amongst various atherogenicity indices, no one was associated with higher incidence risk of mortality either in crude or in adjusted models. For CVD mortality, amongst atherogenicity indices, CI (HR_Q4__vs. Q1_ = 1.61, 95 % CI: 1.07–2.43, P trend = 0.005), CRI-II (HR_Q4__vs. Q1_ = 1.66, 95 % CI: 1.11–2.49, P trend = 0.01) and AIP (HR_Q4__vs. Q1_ = 1.61, 95 % CI: 1.07–2.45, P trend = 0.02) were related to higher incidence risk of CVD-mortality in the crude model. After adjustment for potential confounders, the association remained statistically significance only for CRI-II (HR_Q4__vs. Q1_ = 1.49, 95 % CI: 0.99–2.25, P trend = 0.04) and API (HR_Q4__vs. Q1_ = 1.81, 95 % CI: 1.19–2.74, P trend = 0.01). Regarding non-CVD mortality, CI was inversely related to the risk of mortality after adjustment for potential confounders (HR_Q4__vs. Q1_ = 0.68, 95 % CI: 0.49–0.95, P trend = 0.007).

**Table 4:** Multivariable adjusted HRs for all-cause, CVD, and non-CVD mortality across quartiles of novel atherogenicity indices.

	Case/person years	Model 1	Model 2	Model 3
**All-cause mortality**				
**CI**				
Q1	127/175,516	1	1	1
Q2	124/171,776	1.03 (0.79–1.33)	0.99 (0.76–1.28)	1.01 (0.78–1.32)
Q3	120/173,828	1.06 (0.82–1.37)	0.94 (0.73–1.22)	0.96 (0.74–1.25)
Q4	114/178,685	1.12 (0.87–1.43)	0.90 (0.69–1.61)	0.89 (0.69–1.15)
**P trend^1^**		0.35	0.37	0.33
**CRI-II**				
Q1	131/178,630	1	1	1
Q2	122/171,655	1.07 (0.83–1.39)	1.03 (0.79–1.33)	1.05 (0.81–1.36)
Q3	119/172,879	1.09 (0.85–1.41)	1.07 (0.82–1.38)	1.08 (0.84–1.41)
Q4	114/177,075	1.15 (0.89–1.48)	0.99 (0.77–1.28)	0.99 (0.77–1.28)
**P trend^1^**		0.27	0.97	0.99
**AIP**				
Q1	121/174,394	1	1	1
Q2	125/174,618	0.97 (0.75–1.25)	1.04 (0.80–1.34)	1.01 (0.78–1.30)
Q3	118/174,787	1.02 (0.79–1.31)	1.02 (0.79–1.31)	0.98 (0.76–1.26)
Q4	120/176,440	1.01 (0.78–1.30)	1.15 (0.89–1.48)	1.12 (0.87–1.44)
**P trend^1^**		0.82	0.31	0.44
**CVD mortality**				
**CI**				
Q1	395/175,516	1	1	1
Q2	398/171,776	0.99 (0.62–1.57)	0.96 (0.61–1.52)	0.97 (0.61–1.54)
Q3	268/173,828	1.47 (0.96–2.23)	1.34 (0.88–2.05)	1.38 (0.90–2.10)
Q4	244/178,685	1.61 (1.07–2.43)	1.34 (0.89–2.03)	1.34 (0.88–2.03)
**P trend^1^**		0.005	0.06	0.07
**CRI-II**				
Q1	392/178,630	1	1	1
Q2	333/171,655	1.17 (0.76–1.82)	1.13 (0.73–1.76)	1.16 (0.75–1.81)
Q3	327/172,879	1.19 (0.77–1.84)	1.18 (0.76–1.83)	1.24 (0.80–1.92)
Q4	234/177,075	1.66 (1.11–2.49)	1.47 (0.98–2.21)	1.49 (0.99–2.25)
**P trend^1^**		0.01	0.056	0.04
**AIP**				
Q1	404/174,394	1	1	1
Q2	316/174,618	1.27 (0.82–1.97)	1.38 (0.89–2.14)	1.33 (0.86–2.07)
Q3	310/174,787	1.30 (0.84–2.00)	1.32 (0.85–2.04)	1.25 (0.80–1.94)
Q4	249/176,440	1.61 (1.07–2.45)	1.88 (1.24–2.86)	1.81 (1.19–2.74)
**P trend^1^**		0.02	0.005	0.01
**Non-CVD mortality**				
**CI**				
Q1	187/175,516	1	1	1
Q2	181/171,776	1.04 (0.76–1.43)	1.00 (0.73–1.36)	1.03 (0.75–1.41)
Q3	216/173,828	0.87 (0.62–1.20)	0.75 (0.54–1.05)	0.77 (0.55–1.07)
Q4	213/178,685	0.88 (0.64–1.22)	0.69 (0.50–0.96)	0.68 (0.49–0.95)
**P trend^1^**		0.28	0.01	0.007
**CRI-II**				
Q1	196/178,630	1	1	1
Q2	193/171,655	1.03 (0.74–1.41)	0.97 (0.71–1.34)	0.99 (0.72–1.37)
Q3	187/172,879	1.05 (0.76–1.44)	1.01 (0.73–1.39)	1.01 (0.73–1.39)
Q4	220/177,075	0.89 (0.64–1.23)	0.76 (0.54–1.06)	0.75 (0.53–1.04)
**P trend^1^**	–	0.55	0.13	0.11
**AIP**				
Q1	173/174,394	1	1	1
Q2	208/174,618	0.84 (0.61–1.15)	0.89 (0.65–1.22)	0.86 (0.63–1.19)
Q3	192/174,787	0.90 (0.66–1.23)	0.89 (0.65–1.21)	0.86 (0.63–1.18)
Q4	230/176,440	0.75 (0.54–1.04)	0.85 (0.61–1.17)	0.82 (0.59–1.15)
**P trend^1^**	–	0.13	0.34	0.26

1 Derived from Mantel-Haenszel.

Model 1: crude.

Model 2: Adjusted for age and sex.

Model 3: Additionally adjusted for smoking status, total daily physical activity, marital status, education level.

CI: cholesterol index, CRI: Castelli risk index, and AIP: atherogenic index of plasma.

In stratified analysis by sex **(Supplementary Table 1)**, a strong positive correlation was found between ABSI and the risk of all-cause mortality in females, but not males, after adjusting for confounding variables (HR_Q4__vs. Q1_ = 1.67, 95 % CI:1.14–2.45, P trend = 0.009). None of the anthropometric and atherogenicity indices were associated with CVD risk after adjustment for potential confounders either in males or in females though higher CI scores tended to increase the risk in males (HR_Q4__vs. Q1_ = 1.60, 95 % CI: 0.95–2.68, P trend = 0.05). Regarding non-CVD mortality, a positive significant association was found for ABSI and non-CVD mortality in both males (HR_Q4__vs. Q1_ = 1.75, 95 % CI: 0.90–3.44, P trend<0.001) and females (HR_Q4__vs. Q1_ = 2.34, 95 % CI: 1.46–3.75, P trend<0.001) after adjusting for confounding variables. Furthermore, an increase in the CI index was associated with a decreased risk of non-CVD mortality only in males (HR_Q4__vs. Q1_ = 0.61, 95 % CI:0.38–0.97, P trend = 0.01).

**Supplementary figures 1- 3** illustrate Kaplan-Meier survival analysis curves, demonstrating the occurrence of all-cause, CVD and non-CVD mortality across quartiles of anthropometric and atherogenicity indices. The cumulative survival rate appeared to be greater in the lower quartile of ABSI with all-cause and non-CVD mortality (log rank = 0.001) compared to top quartile of ABSI. There was no apparent difference in the cumulative survival rates for other indicators with all-cause, CVD, and non-CVD, and mortality.

## 4 Discussion

In the present cohort investigation among Iranian adults from the general population, we found that amongst various novel anthropometric and atherogenicity indices, only ABSI, but not others, was directly related to all-cause mortality. In contrast, while no anthropometric indices were linked to the risk of CVD mortality, higher values of CRI-II and AIP, as atherogenicity indices, were associated with increased risk of CVD mortality. In terms of non-CVD mortality, ABSI was linked to higher risk whereas CI was associated with lower risk of non-CVD mortality. Sex-stratified analysis revealed significant direct relation between ABSI and all-cause and non-CVD mortality among females, and CI and non-CVD mortality among males.

Obesity and dyslipidemia, as intertwined risk factors, are of particular importance among NCDs risk factors, with afflicted individuals are at increased risk of cardiovascular events, morbidity and mortality [4]. Understanding the complex relationship between obesity, dyslipidemia, CVD and mortality is an essential step in developing effective prevention and treatment strategies to reduce the burden of NCDs [4]. Obesity, particularly central obesity, is associated with a state of chronic low-grade systemic inflammation with elevated levels of circulating pro-inflammatory cytokines [24]. Although BMI is still the most common metric implemented to determine weight status, it has several drawbacks, and therefore, can sometimes be misleading [7].

ABSI, BRI, and AVI are allometric anthropometric indices which reflects a more accurate representation of the degree of adiposity and associated health risks [25,26]. The advantage of ABSI, BRI, and AVI over BMI is that they determine the amount of visceral adipose tissue and body fat simultaneously [26,27]. ABSI has been showed to perform as a better predictor of CVD and total mortality than other indices such as BMI, WC, and waist-to-hip ratio [[28], [29], [30], [31]].

The findings of the present study regarding a positive association between ABSI and risk of all-cause and non-CVD mortality, but not CVD mortality, were in agreement with a current systematic review and meta-analysis of 24 retrospective cohort studies and 14 cross-sectional studies [29]. They reported that each additional SD in ABSI was associated with an increase in the odds of CVD risk by 21 % and all-cause mortality risk by 55 %. Moreover, in a cohort study, ABSI was independently associated with all-cause mortality risk among older adults. The multivariable-adjusted HRs for all-cause mortality per each SD increment in ABSI was 1.13 (95 % CI: 1.05, 1.21) [27]. Consistently, another population-based cohort study revealed a linear association between ABSI and all-cause mortality among men and women [32]. The same results were declared by another cohort study conducted among a European population [31]. Although in contrast with our findings, these two studies showed a positive linear association between ABSI and CVD mortality [31,32], our earlier cross-sectional analysis on this population also showed a weak association between ABSI and cardiometabolic risk factors and metabolic syndrome [10]. Our results failed to find any significant association between BRI and all-cause, CVD and non-CVD mortality. Inconsistent with our results, recent cohort studies showed a nonlinear association between BRI and all-cause and CVD mortality [33,34].

The positive association of ABSI with the risk of mortality might be explained by its components. As ABSI is formed based on WC, weight and height, higher ABSI indicates that WC is higher than expected for a given height and weight, which in turn, detects a greater fraction of visceral fat [35]. Excess visceral fat could induce a low-grade systemic inflammation by elevating levels of circulating pro-inflammatory cytokines and thus could elevate risk of chronic diseases and mortality [36]. However, further observational and interventional research is needed to authenticate the prediction value of ABSI in mortality.

Novel biomarkers, such as AIP, CRI and CI, recently have been proposed for the prediction of dyslipidemia, atherosclerosis and CVD. These atherogenicity indices are shown to be stronger predictors of CVD risk in comparison with individual lipid risk factors including TC, TG, HDL-C, and LDL-C. AIP is a logarithmic conversion of triglyceride into HDL–C ratio, while CRI is calculated from TC, LDL-C and HDL-C. The results of our study in terms of positive relation between AIP and CVD mortality is aligned with recent cohort studies. Sadeghi et al., showed a significant association between AIP levels and cardiovascular events and its related mortality [37]. Another prospective cohort study among middle-aged and elderly Lithuanian population revealed that higher AIP level was significantly associated with increased CVD mortality in men.

It worth noting that sex-specific association between CI and non-CVD mortality was suggested in the present study. Upon stratifying our outcomes by sex, a significant positive link was observed between CI and non-CVD mortality only in males. We are aware of no earlier study evaluating the relationship of CI and mortality in males and females. However, one possible explanation for this sex-specific association might be due to shorter life expectancy and higher mortality rate in males than females [38]. To explain the sex gap in mortality, lifestyle and biological factors have been invoked. Males have higher rates of risky behaviors including physical inactivity, poor diet, smoking, drug and alcohol use [39]. Available evidence suggests that the biological differences between the sexes, such as genetics and hormones, provide stronger survival resilience to adverse socioeconomic conditions for females than males [39]. In addition, compared with women, men have a more atherogenic lipid profile (higher LDL-C and TG levels) in adulthood [40]. However, more studies are needed to explain the reasons underlying the sex-specific association between CI and mortality. Furthermore, higher values of ABSI were associated with higher risk of all-cause and non-CVD mortality in females, but not in males. It could be explained by lower amount of muscle mass and higher percentage of body fat in females compared to males [41]. A recent systematic review and meta-analysis revealed that higher body fat content was associated with increased all-cause mortality [42].

The positive association between these atherogenicity indices and mortality in the present study could be clarified through some mechanisms. Dyslipidemia, an unbalanced level of blood lipid component, has been proved to induce atherosclerosis by changing the properties of the endothelium, promoting cell adhesion, and inducing the production of monocytes, macrophages, and the proliferation of smooth muscle cells [43]. Atherosclerosis is the main risk factor for CVD, which is the leading cause of mortality worldwide [43].

There are several strengths attributed to the present study. To our knowledge, this is the first longitudinal study that has investigated the relationship between novel anthropometric and atherogenicity indices and mortality from all-cause and CVD and non-CVD in a Middle Eastern country. Large number of participants, the prospective design, face to face interviews to collect data and the long follow-up period were other supremacy of our study. Furthermore, included participants were chosen from both rural and urban areas and thus our findings have good external validity. Despite the mentioned strengths, there are some limitations that could affect our results. Although we controlled several relevant confounding variables, we cannot rule out the possibility of residual confounding by unmeasured or unknown factors. Moreover, we examined the association of baseline values of anthropometric and atherogenicity indices devoid of considering their changes over time which may affect our results.

In summary, we found that novel anthropometric and atherogenicity indices have different potential to predict mortality risk. While all-cause and non-CVD mortality were positively linked to ABSI, as an anthropometric index, CVD mortality were closely and positively related to CRI-II and AIP, as atherogenicity indices Considering our results, it seems that these novel anthropometric and atherogenicity indices which are comprehensive, cheap, accessible and easy to interpret, could be considered for the early identification of subjects at high risk of mortality in both epidemiological studies and clinical practice. Further comprehensive studies which consider the changes of indices over follow-up duration are recommended.

## Data availability statement

The datasets used and/or analyzed during the current study available from the corresponding author on reasonable request.

## Funding

The baseline survey was supported by Grant number 31309304. The Isfahan Cardiovascular Research Center, affiliated to Isfahan University of Medical Sciences funded the biannual follow-ups.

## CRediT authorship contribution statement

**Parisa Hajihashemi:** Writing – original draft. **Noushin Mohammadifard:** Project administration. **Motahare Bateni:** Formal analysis, Methodology. **Fahimeh Haghighatdoost:** Methodology, Project administration. **Maryam Boshtam:** Resources. **Jamshid Najafian:** Resources. **Masoumeh Sadeghi:** Resources. **Niloufar Shabani:** Resources. **Nizal Sarrafzadegan:** Resources.

## Declaration of competing interest

The authors declare that there are no conflicts of interest.

## Appendix Supplementary materials

Image, application 1
