# Supplementary material for: Comparing the association of novel Anthropometric and atherogenicity indices with all-cause, cardiovascular and non-cardiovascular mortality in a general population of Iranian adults
Source: Am J Prev Cardiol. 2025 Jan 27;21:100936. doi: 10.1016/j.ajpc.2025.100936 (PMC11833613; doi:10.1016/j.ajpc.2025.100936)
Supplement: Supplementary file 1 [file mmc1.docx]

**Supplementary Table 1:** multivariate adjusted HRs for All-cause, CVD, and non-CVD mortality across quartiles of anthropometric and atherogenicity indices stratified by sex

|  | |  | **All-cause mortality** | | **CVD-mortality** | | **Non- CVD mortality** | |
| --- | --- | --- | --- | --- | --- | --- | --- | --- |
|  | |  | **Crude** | **Adjusted^1^** | **Crude** | **Adjusted** | **Crude** | **Adjusted** |
| ABSI | Males | Q1 | 1 | 1 | 1 | 1 | 1 | 1 |
|  |  | Q2 | 1.59 (0.94-2.69) | 1.81(1.07-3.06) | 1.62(0.74-3.56) | 1.91(0.87-4.20) | 1.56(0.77-3.16) | 1.74(0.86-3.53) |
|  |  | Q3 | 1.77 (1.07-2.94) | 1.71(1.03-2.86) | 1.69(0.79-3.63) | 1.67(0.77-3.62) | 1.84(0.93-3.62) | 1.75(0.88-3.46) |
|  |  | Q4 | 1.83 (1.11-3.02) | 1.53(0.92-2.55) | 1.53(0.71-3.28) | 1.26(0.58-2.74) | 2.06(1.06-4.02) | 1.75(0.90-3.44) |
|  |  | **P trend^2^** | 0.03 | 0.55 | 0.51 | 0.63 | 0.02 | 0.24 |
|  | Females | Q1 | 1 | 1 | 1 | 1 | 1 | 1 |
|  |  | Q2 | 1.18(0.82-1.71) | 1.08(0.74-1.56) | 0.89(0.49-1.62) | 0.80(0.44-1.45) | 1.42(0.89-2.29) | 1.30(0.81-2.10) |
|  |  | Q3 | 1.48(1.00-2.18) | 1.23(0.83-1.82) | 1.26(0.69-2.30) | 1.03(0.56-1.89) | 1.66(1.00-2.75) | 1.42(0.85-2.36) |
|  |  | Q4 | 2.10(1.44-3.07) | 1.67(1.14-2.45) | 1.08(0.54-2.18) | 0.88(0.43-1.77) | 2.94(1.84-4.71) | 2.34(1.46-3.75) |
|  |  | **P trend^2^** | <0.001 | 0.009 | 0.57 | 0.87 | <0.001 | <0.001 |
| AVI | Males | Q1 | 1 | 1 | 1 | 1 | 1 | 1 |
|  |  | Q2 | 0.88(0.64-1.23) | 0.87(0.63-1.22) | 0.94(0.55-1.59) | 0.93(0.55-1.57) | 0.85(0.56-1.30) | 0.84(0.55-1.29) |
|  |  | Q3 | 0.93(0.67-1.29) | 0.90(0.64-1.25) | 1.13(0.68-1.88) | 1.08(0.65-1.81) | 0.81(0.52-1.25) | 0.78(0.50-1.21) |
|  |  | Q4 | 1.25(0.90-1.74) | 1.12(0.8-1.57) | 1.35(0.79-2.28) | 1.16(0.67-1.99) | 1.20(0.78-1.83) | 1.09(0.71-1.68) |
|  |  | **P trend^2^** | 0.21 | 0.56 | 0.21 | 0.48 | 0.56 | 0.86 |
|  | Females | Q1 | 1 | 1 | 1 | 1 | 1 | 1 |
|  |  | Q2 | 0.93(0.61-1.42) | 0.89(0.58-1.36) | 1.08(0.53-2.22) | 1.00(0.49-2.06) | 0.86(0.51-1.44) | 0.83(0.49-1.40) |
|  |  | Q3 | 0.86(0.56-1.31) | 0.75(0.49-1.15) | 1.06(0.52-2.15) | 0.94(0.46-1.92) | 0.76(0.45-1.29) | 0.67(0.39-1.13) |
|  |  | Q4 | 0.91 (0.62-1.35) | 0.82(0.55-1.22) | 0.98(0.50-1.94) | 0.87(0.44-1.72) | 0.88(0.54-1.42) | 0.79(0.49-1.28) |
|  |  | **P trend^2^** | 0.65 | 0.30 | 0.90 | 0.63 | 0.64 | 0.34 |
| BRI | Males | Q1 | 1 | 1 | 1 | 1 | 1 | 1 |
|  |  | Q2 | 1.22(0.90-1.64) | 1.06(0.79-1.44) | 1.23(0.76-1.99) | 1.05(0.64-1.71) | 1.21(0.83-1.77) | 1.07(0.73-1.58) |
|  |  | Q3 | 1.29(0.94-1.79) | 1.04(0.75-1.44) | 1.52(0.92-2.51) | 1.19(0.72-1.99) | 1.16(0.76-1.76) | 0.94(0.61-1.44) |
|  |  | Q4 | 1.81(1.21-2.68) | 1.02(0.68-1.53) | 2.30(1.27-4.15) | 1.26(0.69-2.31) | 1.50(0.88-2.58) | 0.87(0.50-1.50) |
|  |  | **P trend^2^** | 0.005 | 0.87 | 0.006 | 0.35 | 0.18 | 0.57 |
|  | Females | Q1 | 1 | 1 | 1 | 1 | 1 | 1 |
|  |  | Q2 | 0.82(0.47-1.43) | 0.79(0.46-1.39) | 0.78(0.28-2.15) | 0.77(0.28-2.14) | 0.84(0.43-1.62) | 0.80(0.41-1.55) |
|  |  | Q3 | 0.96(0.59-1.57) | 0.71(0.43-1.16) | 1.39(0.6-3.26) | 1.03(0.44-2.42) | 0.77(0.42-1.42) | 0.57(0.30-1.05) |
|  |  | Q4 | 1.22(0.78-1.93) | 0.76(0.48-1.21) | 1.45(0.64-3.27) | 0.88(0.39-2.01) | 1.12(0.65-1.95) | 0.70(0.4-1.23) |
|  |  | **P trend^2^** | 0.09 | 0.38 | 0.13 | 0.91 | 0.33 | 0.30 |
|  | Males | Q1 | 1 | 1 | 1 | 1 | 1 | 1 |
|  |  | Q2 | 1.03 (0.75-1.41) | 1.10 (0.80-1.53) | 0.93(0.53-1.63) | 0.95(0.54-1.69) | 1.70(1.07-2.70) | 1.19(0.80-1.76) |
| CI |  | Q3 | 0.92 (0.66-1.28) | 1.06 (0.76-1.48) | 1.39(0.83-2.31) | 1.58(0.94-2.64) | 1.84(1.16-2.92) | 0.79(0.50-1.23) |
|  |  | Q4 | 0.81 (0.57-1.13) | 0.84 (0.59-1.19) | 1.24(0.73-2.09) | 1.27(0.75-2.16) | 1.16(0.70-1.93) | 0.61(0.38-0.97) |
|  |  | **P trend^2^** | 0.18 | 0.35 | 0.21 | 0.15 | 0.006 | 0.01 |
|  | Females | Q1 | 1 | 1 | 1 | 1 | 1 | 1 |
|  |  | Q2 | 1.06(0.68-1.65) | 0.86(0.55-1.34) | 1.16(0.52-2.60) | 0.97(0.43-2.18) | 0.70(0.43-1.13) | 0.82(0.48-1.40) |
|  |  | Q3 | 1.37(0.91-2.08) | 0.80(0.53-1.22) | 1.73(0.83-3.62) | 1.04(0.49-2.18) | 0.71(0.44-1.15) | 0.72(0.43-1.20) |
|  |  | Q4 | 1.74(1.17-2.58) | 0.86(0.58-1.29) | 2.52(1.26-5.04) | 1.23(0.61-2.48) | 0.86(0.55-1.35) | 0.70(0.43-1.15) |
|  |  | **P trend^2^** | 0.002 | 0.54 | 0.003 | 0.45 | 0.10 | 0.16 |
|  | Males | Q1 | 1 | 1 | 1 | 1 | 1 | 1 |
|  |  | Q2 | 1.06(0.76-1.48) | 1.12(0.80-1.57) | 1.08(0.62-1.89) | 1.16(0.66-2.04) | 1.04(0.69-1.59) | 1.09(0.72-1.67) |
|  |  | Q3 | 1.09(0.78-1.53) | 1.24(0.88-1.73) | 1.19(0.69-2.05) | 1.38(0.79-2.39) | 1.04(0.68-1.59) | 1.16(0.76-1.78) |
|  |  | Q4 | 1.05(0.76-1.47) | 1.08(0.77-1.51) | 1.55(0.93-2.57) | 1.60(0.95-2.68) | 0.78(0.49-1.22) | 0.78(0.50-1.24) |
| CRI-II |  | **P trend^2^** | 0.69 | 0.53 | 0.07 | 0.05 | 0.33 | 0.42 |
|  | Females | Q1 | 1 | 1 | 1 | 1 | 1 | 1 |
|  |  | Q2 | 1.13(0.75-1.69) | 0.90(0.60-1.36) | 1.37(0.67-2.81) | 1.01(0.49-2.08) | 1.02(0.62-1.68) | 0.86(0.52-1.41) |
|  |  | Q3 | 1.13(0.76-1.70) | 0.86(0.57-1.29) | 1.26(0.61-2.59) | 0.92(0.44-1.90) | 1.08(0.67-1.76) | 0.83(0.51-1.35) |
|  |  | Q4 | 0.31(0.89-1.93) | 0.83(0.56-1.23) | 1.92(0.99-3.75) | 1.19(0.60-2.32) | 1.05(0.64-1.71) | 0.67(0.41-1.09) |
|  |  | **P trend^2^** | 0.18 | 0.36 | 0.06 | 0.61 | 0.77 | 0.11 |
|  | Males | Q1 | 1 | 1 | 1 | 1 | 1 | 1 |
|  |  | Q2 | 0.73(0.52-1.02) | 0.87(0.62-1.23) | 0.83(0.47-1.46) | 1.03(0.59-1.81) | 0.68(0.44-1.03) | 0.80(0.52-1.22) |
|  |  | Q3 | 0.84(0.61-1.15) | 1.01(0.73-1.39) | 1.14(0.68-1.92) | 1.36(0.81-2.30) | 0.69(0.45-1.04) | 0.83(0.55-1.26) |
| AIP |  | Q4 | 0.63(0.45-0.88) | 0.93(0.67-1.30) | 0.95(0.56-1.60) | 1.41(0.83-2.38) | 0.47(0.30-0.73) | 0.70(0.44-1.09) |
|  |  | **P trend^2^** | 0.019 | 0.88 | 0.85 | 0.12 | 0.001 | 0.14 |
|  | Females | Q1 | 1 | 1 | 1 | 1 | 1 | 1 |
|  |  | Q2 | 1.42(0.95-2.13) | 1.17(0.78-1.76) | 2.39(1.14-5.03) | 1.88(0.89-3.95) | 1.11(0.68-1.81) | 0.94(0.58-1.55) |
|  |  | Q3 | 1.32(0.87-1.99) | 0.93(0.61-1.40) | 1.52(0.67-3.42) | 1.00(0.44-2.26) | 1.25(0.77-2.03) | 0.91(0.56-1.48) |
|  |  | Q4 | 1.80(1.21-2.68) | 1.38(0.92-2.06) | 3.39(1.64-7.01) | 2.39(1.15-4.95) | 1.29(0.78-2.12) | 1.00(0.61-1.65) |
|  |  | **P trend^2^** | 0.008 | 0.24 | 0.004 | 0.07 | 0.25 | 0.98 |
|  |  |  |  |  |  |  |  |  |

^1^ Adjusted for age, smoking status, total daily physical activity, marital status, education level.

2 Derived from Mantel-Haenszel.

ABSI: a body shape index, AVI: abdominal volume index, BRI: body roundness index, CI: cholesterol index, CRI: Castelli risk index, and AIP: atherogenic index of plasma


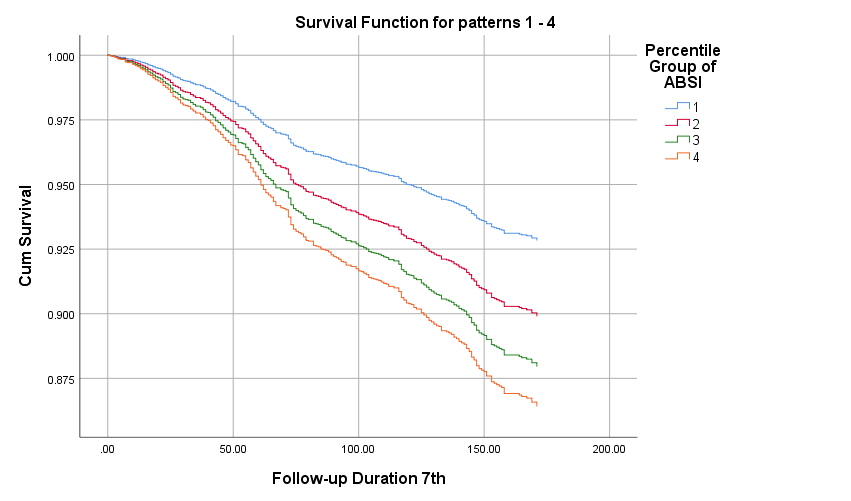

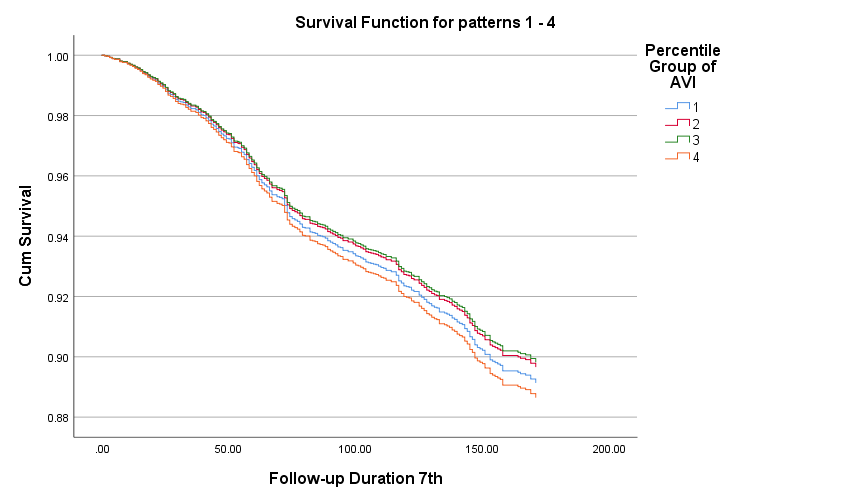


Times (years)

0 5 10 15 20

0 5 10 15 20

Log Rank<0.001

Log Rank: 0.80

Times (years)

Cumulative survival

Cumulative survival

A1: ABSI A2: AVI


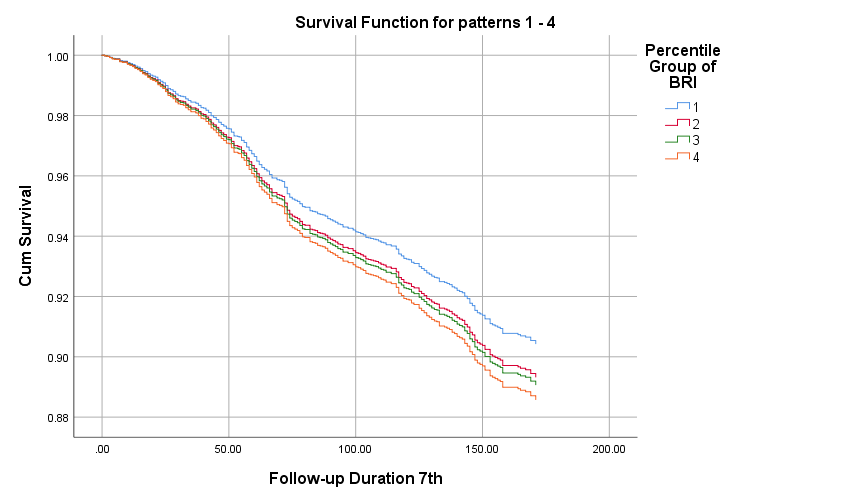

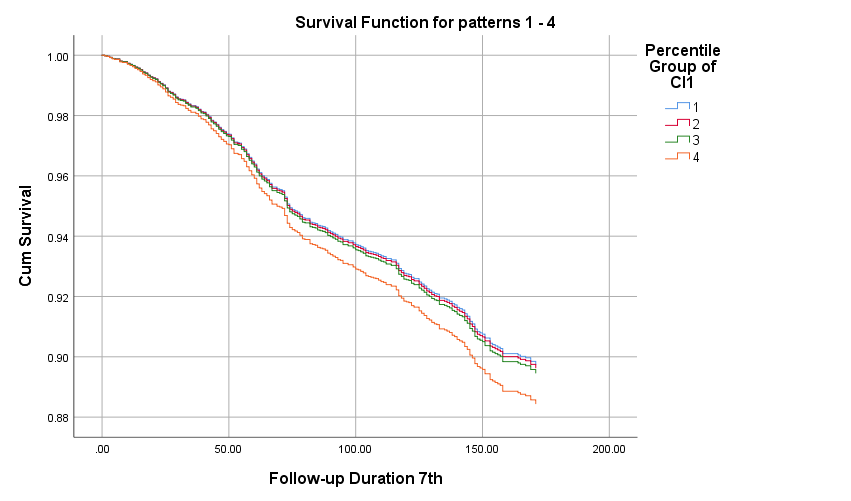


0 5 10 15 20

0 5 10 15 20

Log Rank: 0.581

Log Rank: 0.759

Cumulative survival

Times (years)

Times (years)

Cumulative survival

A3: BRI B1: CI


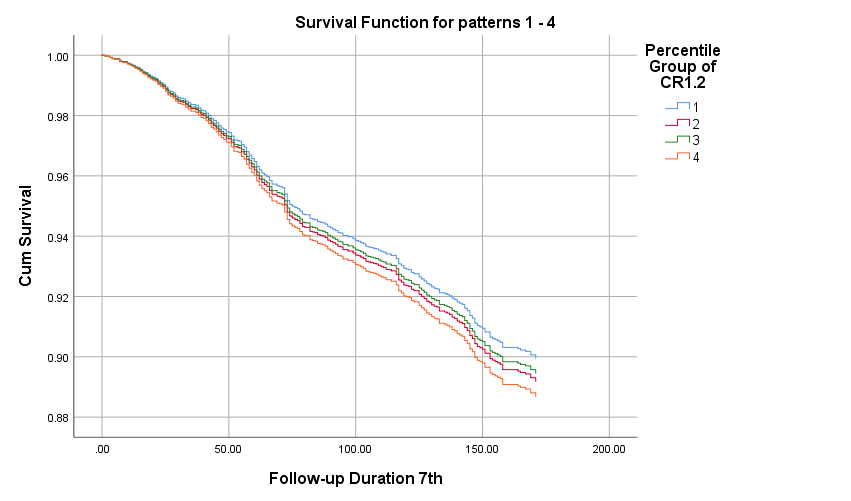


Quartile 1

Quartile 2

Quartile 3

Quartile 4

0 5 10 15 20

Log Rank: 0.815

Cumulative survival

Times (years)

Supplementary Figure 1

B2: CRI.II


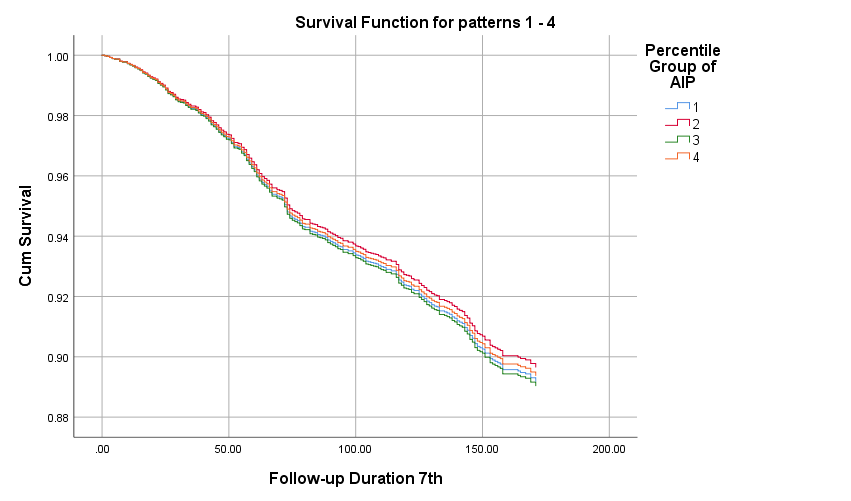


0 5 10 15 20

Log Rank: 0.971

Cumulative survival

Times (years)

B3: AIP


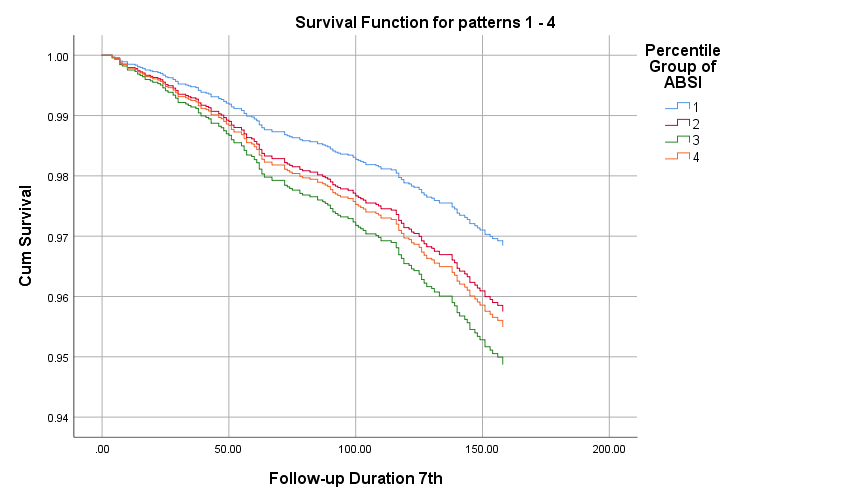

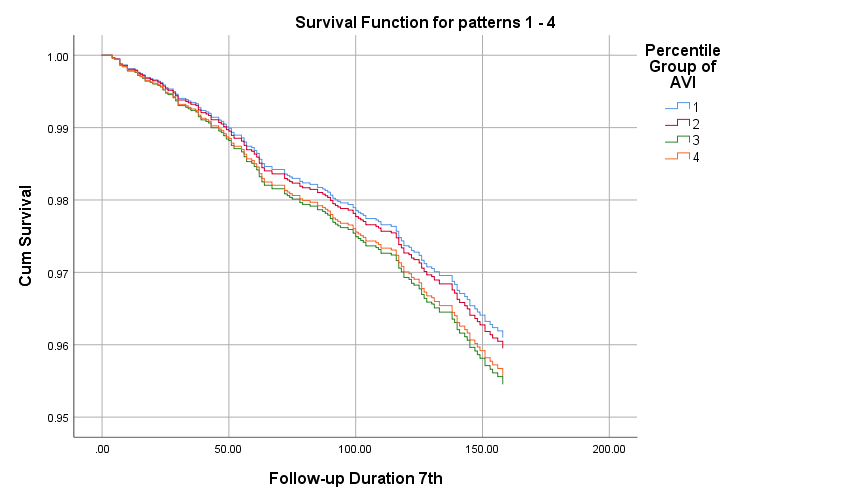


A1: ABSI

Times (years)

A2: AVI

0 5 10 15 20

Times (years)

0 5 10 15 20

Log Rank: 0.169

Log Rank: 0.875

Cumulative survival

Cumulative survival


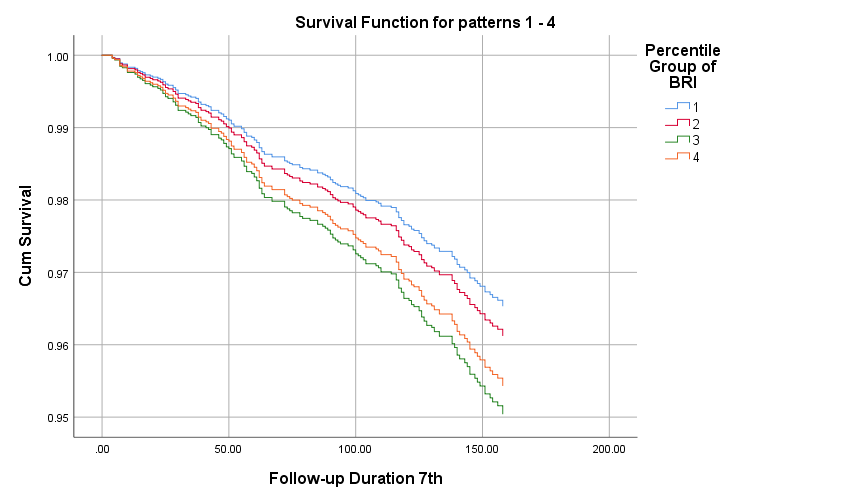

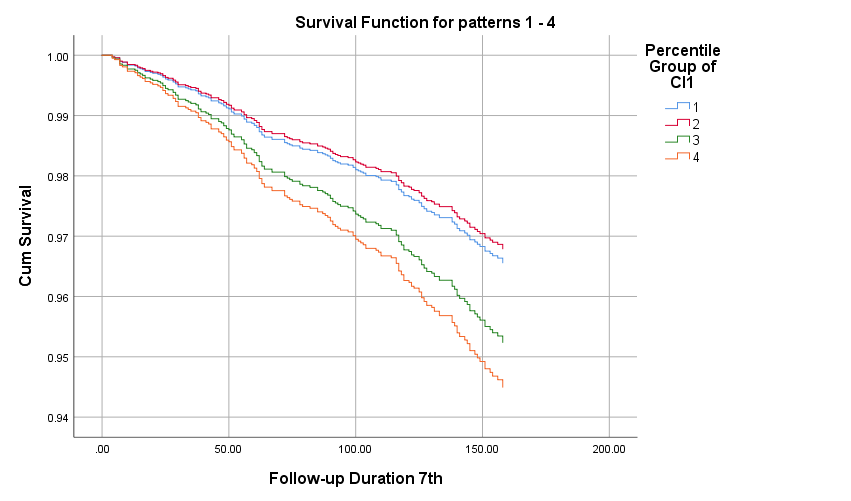


B1: CI

0 5 10 15 20

A3: BRI

0 5 10 15 20

Log Rank: 0.342

Log Rank: 0.469

Times (years)

Times (years)

Cumulative survival

Cumulative survival


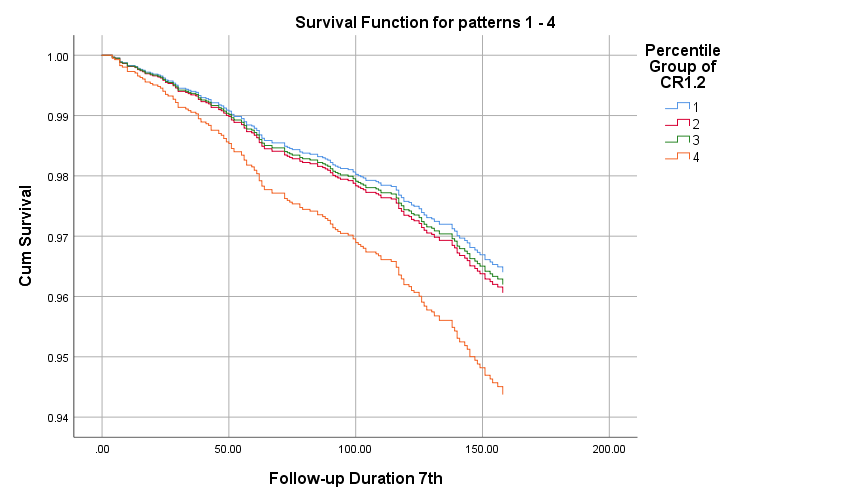


Quartile 1

Quartile 2

Quartile 3

Quartile 4

Supplementary Figure 2

Times (years)

0 5 10 15 20

Log Rank: 0.084

Cumulative survival

B2: CRI.II


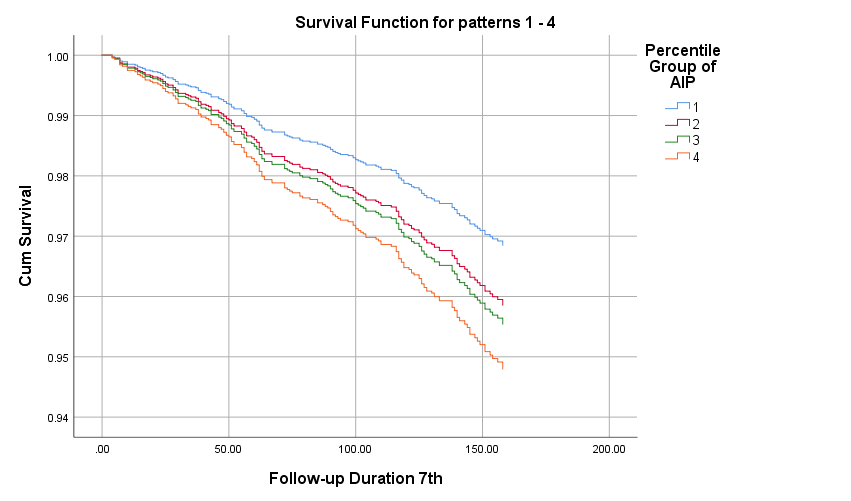


0 5 10 15 20

Log Rank: 0.143

Cumulative survival

Times (years)

B3: AIP


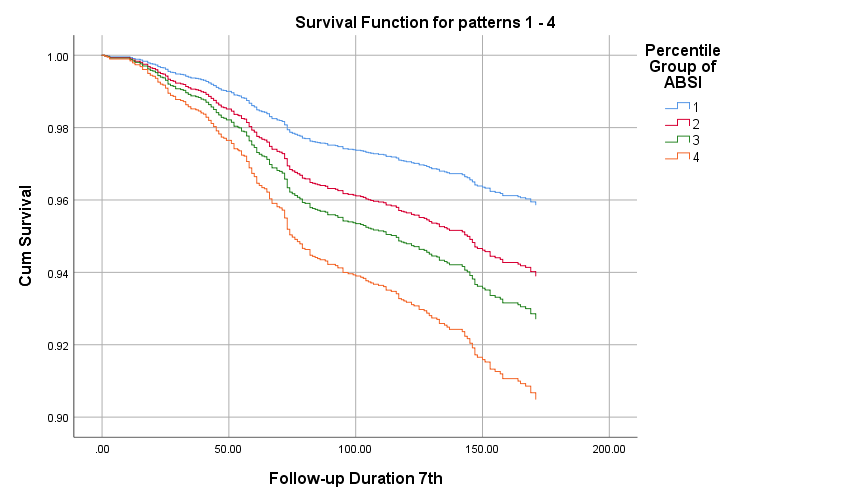


Times (years)

0 5 10 15 20

Log Rank<0.001

Cumulative survival

Cumulative survival

A1: ABSI


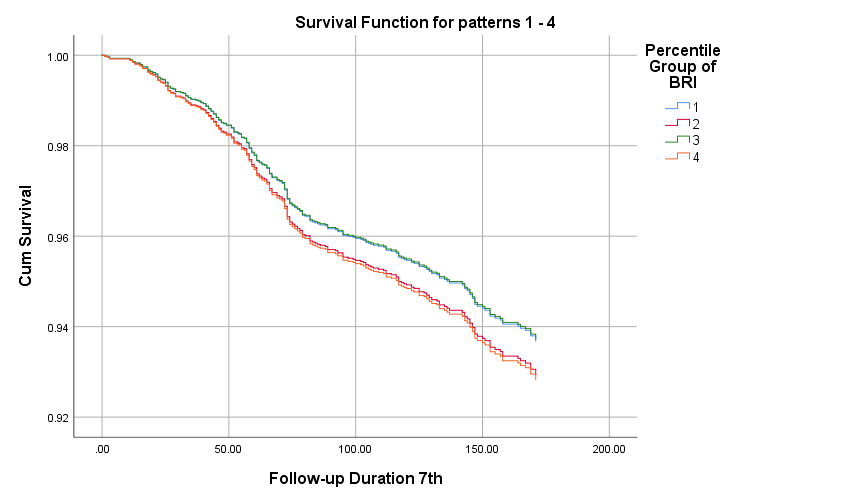


0 5 10 15 20

Log Rank: 0.771

Cumulative survival

Cumulative survival

Times (years)

A3: BRI


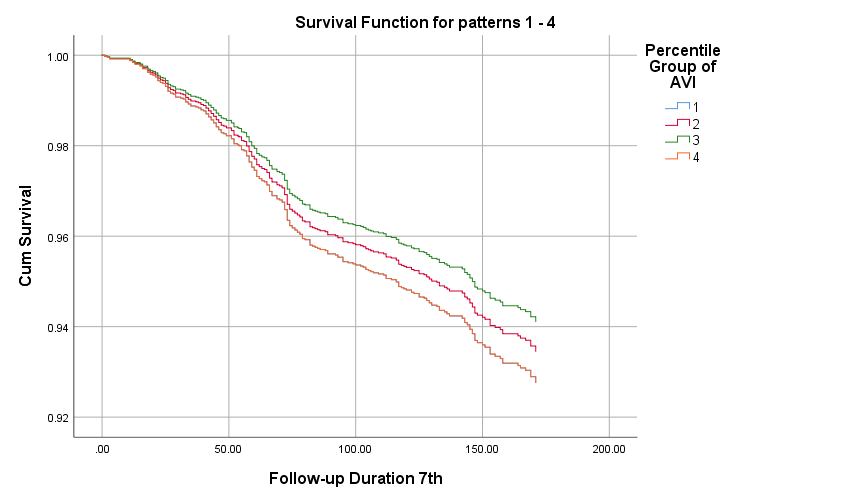


0 5 10 15 20

Log Rank: 0.562

Times (years)

A2: AVI


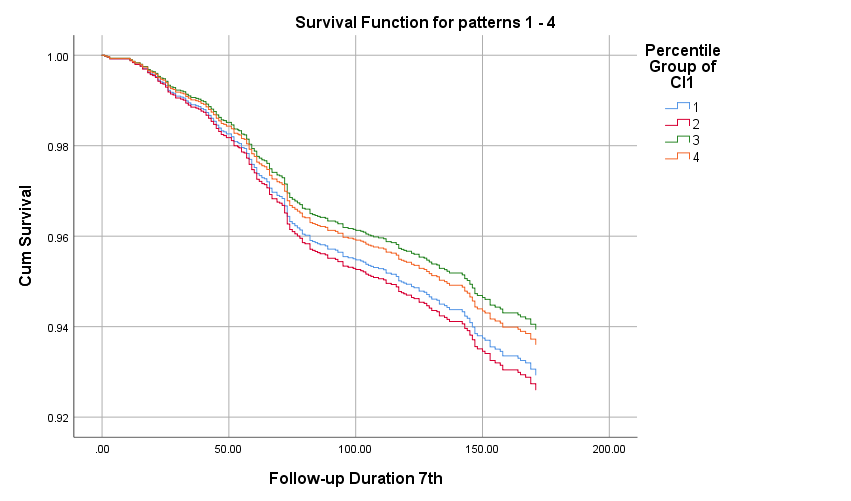


0 5 10 15 20

Log Rank: 0.195

Times (years)

B1: CI


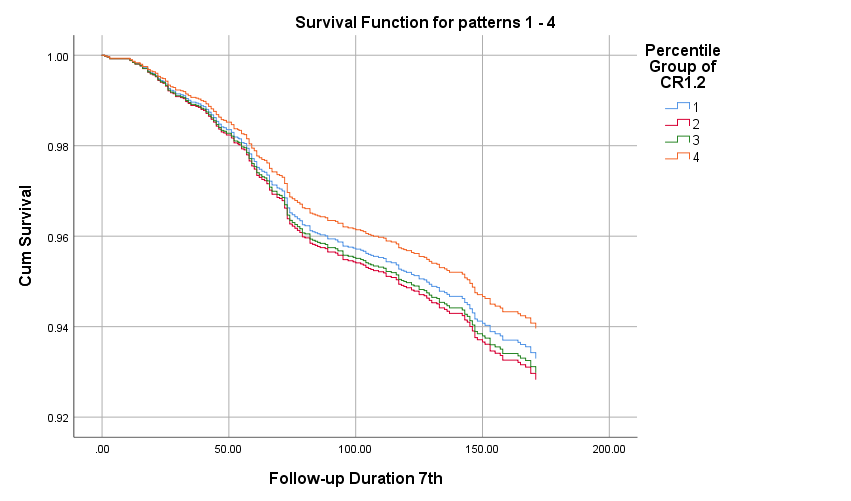


Quartile 1

Quartile 2

Quartile 3

Quartile 4

Times (years)

0 5 10 15 20

Log Rank: 0.246

Cumulative survival

Supplementary Figure 3

B2: CRI.II


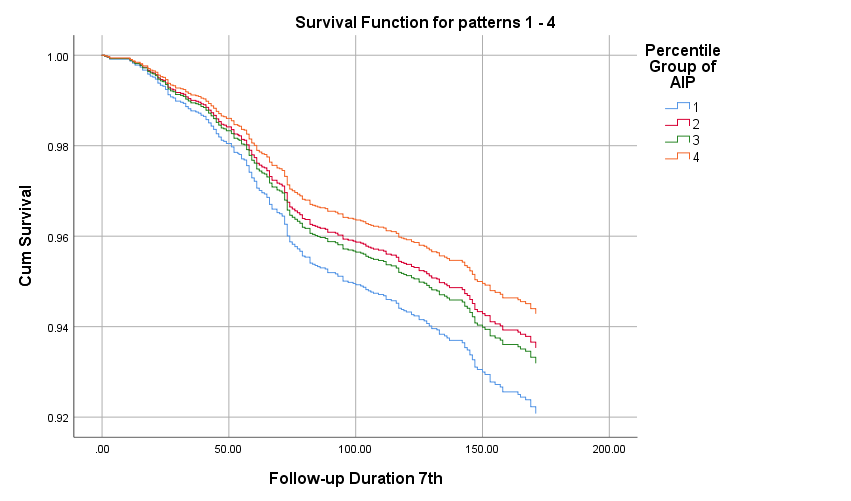


Times (years)

0 5 10 15 20

Log Rank: 0.246

Cumulative survival

B3: AIP

**Supplementary Figure 1:** Kaplan–Meier curves to estimate cumulative hazard for all-cause mortality by quartiles of anthropometric (A1= a body shape index (ABSI), A2= abdominal volume index (AVI), and A3= body roundness index (BRI)) and atherogenicity (B1= cholesterol index (CI), B2= Castelli risk index (CRI-II), B3= atherogenic index of plasma (AIP)) indices.

**Supplementary Figure 2:** Kaplan–Meier curves to estimate cumulative hazard for CVD mortality by quartiles of anthropometric (A1= a body shape index (ABSI), A2= abdominal volume index (AVI), and A3= body roundness index (BRI)) and atherogenicity (B1= cholesterol index (CI), B2= Castelli risk index (CRI-II), B3= atherogenic index of plasma (AIP)) indices.

**Supplementary Figure 3:** Kaplan–Meier curves to estimate cumulative hazard for non-CVD mortality by quartiles of anthropometric (A1= a body shape index (ABSI), A2= abdominal volume index (AVI), and A3= body roundness index (BRI)) and atherogenicity (B1= cholesterol index (CI), B2= Castelli risk index (CRI-II), B3= atherogenic index of plasma (AIP)) indices.
